# Role of NLRP3 in the metabolism of bile acids and gut microbiota in CCl4-induced liver fibrosis

**DOI:** 10.1128/spectrum.00148-25

**Published:** 2025-07-21

**Authors:** Shu Feng, Tao Ran, Xingming Xie, Xueke Zhao

**Affiliations:** 1 Department of Infectious Diseases, The Affiliated Hospital of Guizhou Medical University 74720, Guiyang, Guizhou, People's Republic of China; 2 Department of Medical Examination Center, The Affiliated Hospital of Guizhou Medical University 74720, Guiyang, Guizhou, People’s Republic of China; 3 Guizhou Institute of Precision Medicine, The Affiliated Hospital of Guizhou Medical University, Guiyang, Guizhou, People’s Republic of China; Geisel School of Medicine at Dartmouth, Lebanon, New Hampshire, USA

**Keywords:** liver fibrosis, NLRP3, bile acid, gut microbiota

## Abstract

NOD-like receptor protein 3 (NLRP3) is an important pro-fibrosis factor in the liver. Abnormal bile acid (BA) metabolism and gut dysbiosis are also involved in liver fibrosis. However, the role of NLRP3 in the metabolism of bile acids and gut microbiota in liver fibrosis is unclear, which contributes to the understanding of the mechanism of NLRP3 in liver fibrosis. The male C57BL/6 J mice were divided into the following groups: (i) control group (ii); CCl4 group (iii); NLRP3^−/^− + CCl4 group (iv); NLRP3^−/^− group. The interventions were delivered three times per week for 12 weeks. Liver tissues and intestinal contents were collected. Targeted metabolomics was performed to determine BAs in liver tissues. The 16S rRNA sequencing was adopted to explore the gut microbiota. Total BAs, primary BAs, and secondary BAs were decreased in the CCl4 group compared to the control group, and NLRP3 knockout restored the CCl4-induced BAs disorder. The increased 7α-hydroxy-4-cholesten-3-one (C4) was observed in the CCl4 group, and NLRP3 knockout decreased the C4 level in CCl4-induced fibrosis. Additionally, the levels of C4 and BAs showed no significant alterations in the NLRP3^−/−^ group compared with the control group. Compared to the control group, 16S rRNA sequencing revealed the NLRP3^−/−^ group exhibited increased intestinal alpha diversity and altered beta diversity. Additionally, we observed changes in signaling pathway activity and gut microbiota-derived ECs. 16S rRNA sequencing revealed differential distribution of gut microbiota among the control, CCl4, and NLRP3^−/−^ +CCl4 mice. The linear discriminant analysis (LDA) coupled with effect size (LEfSe) analysis showed that the abundances of Aeromonas, Akkermansia, and Dehalobacterium increased in the gut of CCl4 group and decreased in NLRP3^−/−^ +CCl4 mice at the genus level. The abundances of Allobaculum, Odoribacter, and Ruminococcus decreased in the gut of CCl4 group and increased in NLRP3^−/−^ +CCl4 mice. The abundances of Alistipes, Bacteroides, Clostridium, Lactococcus, Parabacteroides, and Pseudoramibacter_Eubacterium were significantly increased in NLRP3^−/−^ +CCl4 mice. LPS increased in the CCl4 group and decreased after NLRP3 knockout. Lipopolysaccharides (LPS) had a positive correlation with Bacteroides, Parabacteroides, Aeromonas, and Dehalobacterium and a negative correlation with Allobaculum. Some important signaling pathways (e.g., dopaminergic synapse, serotonergic synapse, non-homologous end-joining, basal transcription factors, and isoflavonoid biosynthesis) and enzymes (e.g., Enzyme Commission (EC) number 5.2.1.4, EC 1.4.3.19, EC 3.4.24.20, EC 1.14.13.1, EC 2.3.3.5, EC 1.14.16.1, EC 1.3.8.7, EC 4.2.1.119 , and EC 5.3.3.17) were upregulated in the CCl4 group and reversed by NLRP3 knockout. Correlation analyses revealed that liver BAs were more closely related to some gut microbiota and LPS. The decreased BA level in CCl4-induced liver fibrosis reflected liver injury. NLRP3 knockout restored the BA level, and NLRP3 knockout protected liver from injury. Increased liver LPS reflected bacterial translocation into the liver. NLRP3 knockout decreased liver LPS level and ameliorated CCl4-induced gut microbiota dysregulation. Taken together, NLRP3 damaged the liver and reduced BAs production and might induce bacterial translocation into the liver to promote liver fibrosis.

**IMPORTANCE:**

NOD-like receptor protein 3 (NLRP3) is an important pro-fibrosis factor in the liver. Our previous study also demonstrated that knockout of NLRP3 can alleviate liver fibrosis. In the current study, we discovered NLRP3 knockout restored the BA level and protected liver from injury. NLRP3 knockout decreased the liver LPS level and ameliorated CCl4-induced gut microbiota dysregulation, and some beneficial bacteria were identified. The current study contributes to the understanding of the mechanism of NLRP3 in liver fibrosis and may provide intervention methods for liver fibrosis caused by damage-associated molecular patterns.

## INTRODUCTION

Activation of the NLRP3 inflammasome occurs in liver fibrosis. Inhibiting the NLRP3 inflammasome alleviated liver damage, inflammation, and fibrosis in mice (1, 2). Our previous study also demonstrated that the NLRP3 inflammasome signaling pathway plays an important role in the development and progression of liver fibrosis and that knockout of NLRP3 can alleviate liver fibrosis (3). The occurrence of liver fibrosis can lead to changes in bile acid metabolism, and the gut microbiota is closely related to the bile acid metabolism. After the primary conjugated bile acids enter the intestine from the liver, they are metabolized into secondary bile acids under the action of bacteria. And the BA levels are also related to the growth of gut bacteria. The balance of BAs *in vivo* is tightly regulated by gut-liver signal transduction. Due to the existence of the liver-gut axis, numerous studies have shown that changes in hepatic bile acids and gut microbiota are closely related to the occurrence and development of liver fibrosis (4–7). Furthermore, bile acids can activate the NLRP3 inflammasome to induce liver fibrosis (8–10). However, the effects of NLRP3 on bile acid metabolism in the liver and gut microbiota remain unclear. Therefore, it is important to investigate the role of NLRP3 on hepatic bile acid metabolism and gut microbiota to discover underlying treatment strategies for liver fibrosis.

In the current study, a CCl4-induced liver fibrosis model and NLRP3^−/−^ mice were established to investigate the effects of NLRP3 on hepatic bile acid metabolism and gut microbiota. Targeted metabolomics was performed to determine BA level in liver tissue. 16S rRNA sequencing has been used to study the gut microbiota. The differential liver bile acids, gut microbiota, and altered signaling pathways were examined to find out the role of NLRP3 in liver fibrosis.

## MATERIALS AND METHODS

### Mice

Male C57BL/6 J mice weighing 20 ± 3 g at 7 weeks of age were purchased from Beijing Vital River Laboratory Animal Technology Co. Ltd. (Beijing, China), and Shanghai Model Organisms Center, Inc. provided the NLRP3-deficient mice (Shanghai, China). NLRP3 gene knockout mice were created as previously described (3).

### Mouse model

The mouse model was used as previously described (3). The male C57BL/6 J mice (7 weeks old, weighing 20 ± 3 g) were divided into the following groups: (i) control group: intraperitoneal injection of corn oil (ii); CCl4 group: 20% CCl4 corn oil solution injected intraperitoneally at 5 µL/g body weight (iii); NLRP3^−/−^ + CCl4 group: 20% CCl4 intraperitoneal injection into NLRP3 knockout mice (C57BL/6J background), using the same method as the CCl4 group (iv). NLRP3^−/−^ group: NLRP3 knockout mice with intraperitoneal injection of corn oil.

Interventions were delivered three times per week for 12 weeks (*n* = 4). At the end of the experiment, liver tissue and intestinal contents of the mice were collected under sterile conditions.

All mice were maintained under specific pathogen-free (SPF) conditions and fed a standardized irradiated chow (XTI01WC-009, Jiangsu Synergetic Medical Bioengineering Co., Ltd, China) *ad libitum* to prevent microbiome contamination. Autoclaved water was provided in sterile bottles and refreshed twice weekly. Animals were housed in ventilated cages containing autoclaved corn cob bedding (changed weekly) under controlled environmental conditions (12 h light/dark cycle, 20°C–22°C, 45±5% humidity). Cage density was maintained at ≤5 mice/cage to minimize stress-induced microbiota alterations. Littermates were used to stabilize baseline microbiota. The animal study was approved by the Animal Ethics Committee of the Hospital Affiliated with Guizhou Medical University (No. 2000732).

### Hematoxylin and eosin (H&E) and Masson’s trichrome staining

H&E staining was performed using a commercial kit (G1120, Solarbio Biotechnology Co., Ltd., Beijing, China), and Masson’s trichrome staining was carried out with another kit (G1340, Solarbio Biotechnology Co., Ltd.). All procedures strictly followed the manufacturer’s protocols.

### Protein extraction and Western blot analysis

Total proteins were extracted using RIPA buffer containing protease inhibitors, and protein concentrations were measured using a BCA kit (Solarbio, Beijing, China). The proteins were separated by 10% SDS-PAGE and transferred onto a nitrocellulose membrane. The membrane was blocked with 5% non-fat milk for 1 h at room temperature, followed by incubation with primary antibodies overnight at 4°C. Primary antibodies included GAPDH (1:1000, 2118, CST), Collagen I antibody (1:1000, ab260043, abcam), and α-SMA (1:5000, 14395-1-AP, Proteintech). Subsequently, the membrane was incubated with a secondary antibody for 1 h at room temperature. The signals were detected using a sensitive enhanced chemiluminescence (ECL) detection kit (PK10002, Proteintech).

### Targeted metabolomics for BAs

The BA concentration in the samples was quantified using ultraperformance liquid chromatography coupled with triple quadrupole mass spectrometry (UPLC-TQMS, Waters, Milford, MA) conducted by Metabo-Profile Biotechnology Co., Ltd.(Shanghai, China). BAs detected in the liver include T-w-MCA, T-α-MCA, T-β-MCA, THDCA, w-MCA, α-MCA, β-MCA, HCA, AlloCA, NorCA, muroCA, HDCA, β-DCA, isoDCA, NorDCA, isoalloLCA, isoLCA, dehydroLCA, 6-KetoLCA, 7-KetoLCA, 12-KetoLCA, apoCA, 6,7-DiketoLCA, 7-DHCA, 12-DHCA, and UDCA-7S. The C4 was also detected.

### Terminal ileum microbiota assessment

Total genomic DNA samples were extracted using the OMEGA Soil DNA Kit (M5635-02) (Omega Bio-Tek, Norcross, GA, USA) according to the manufacturer’s instructions. The hypervariable region V3–V4 of 16S rRNA was chosen as the PCR-amplified region. The bacterial forward primer was 5′- ACTCCTACGGGAGGCAGCA −3′, and the reverse primer was 5′-GGACTACHVGGGTWTCTAAT −3′. The PCR conditions were set as follows: initial denaturation at 98°C for 2 minutes, followed by 25 cycles consisting of denaturation at 98°C for 15 seconds, annealing at 55°C for 30 seconds, and extension at 72°C for 30 seconds, with a final extension of 5 minutes at 72°C, and hold at 10°C. The PCR products were purified and quantified. Subsequently, paired-end 2 × 250 bp sequencing was performed using the Illumina NovaSeq platform with the NovaSeq 6000 SP reagent Kit (500 cycles) and was conducted by Metabo-Profile Biotechnology Co., Ltd. (Shanghai, China). Sequence analysis was performed using QIIME2 (2019.4) with slight modification according to the official tutorials (https://docs.qiime2.org/2019.4/tutorials/) for operational taxonomic unit (OTU) annotation.

### Measurement of LPS concentration

Liver LPS concentration was measured using the Mouse LPS ELISA Kit (CSB-E13066m, Houston, TX, USA) according to the manufacturer’s instructions.

### Statistical analysis

#### Targeted metabolomics data analysis

The Kolmogorov-Smirnov test determined the normality of the distribution of BA concentrations. Two-tailed Student’s t-test was performed on the normally distributed continuous data. Non-normally distributed continuous data were analyzed using the Mann-Whitney test. A *P* value < 0.05 was considered statistically significant.

#### Sequencing data analysis

(i) Based on the index and barcode information, the original sequences were divided into libraries and samples, and barcode sequences were removed (ii). Vsearch software was used for OTU clustering of sequences. OTU tables were obtained (iii). The alpha diversity level (Chao1, Simpson, and Shannon indices) of each sample was assessed using the OTU tables (iv). According to OTU tables, principal coordinates analysis (PCoA) is based on the distance matrix of each sample to measure the difference and significance of beta diversity between different groups (v). According to the species taxonomy, the LDA effect size (LEfSe, LDA ≥ 3.0, *P* value < 0.05) was conducted for differential microbiota screening (vi). Functional difference analyses (KEGG pathway and EC Enzyme) were performed based on the gene number tables at different taxonomic levels. One-way ANOVA or Kruskal-Wallis test was performed to compare differences in KEGG pathways and EC enzymes among three groups (*P* value < 0.05).

#### Spearman correlation analysis

The correlations between BAs or LPS and the microbial abundance were analyzed and visualized via Oebiotech Cloud (https://cloud.oebiotech.com/). A *P* value < 0.05 was considered statistically significant.

## RESULTS

**Fig 1:**
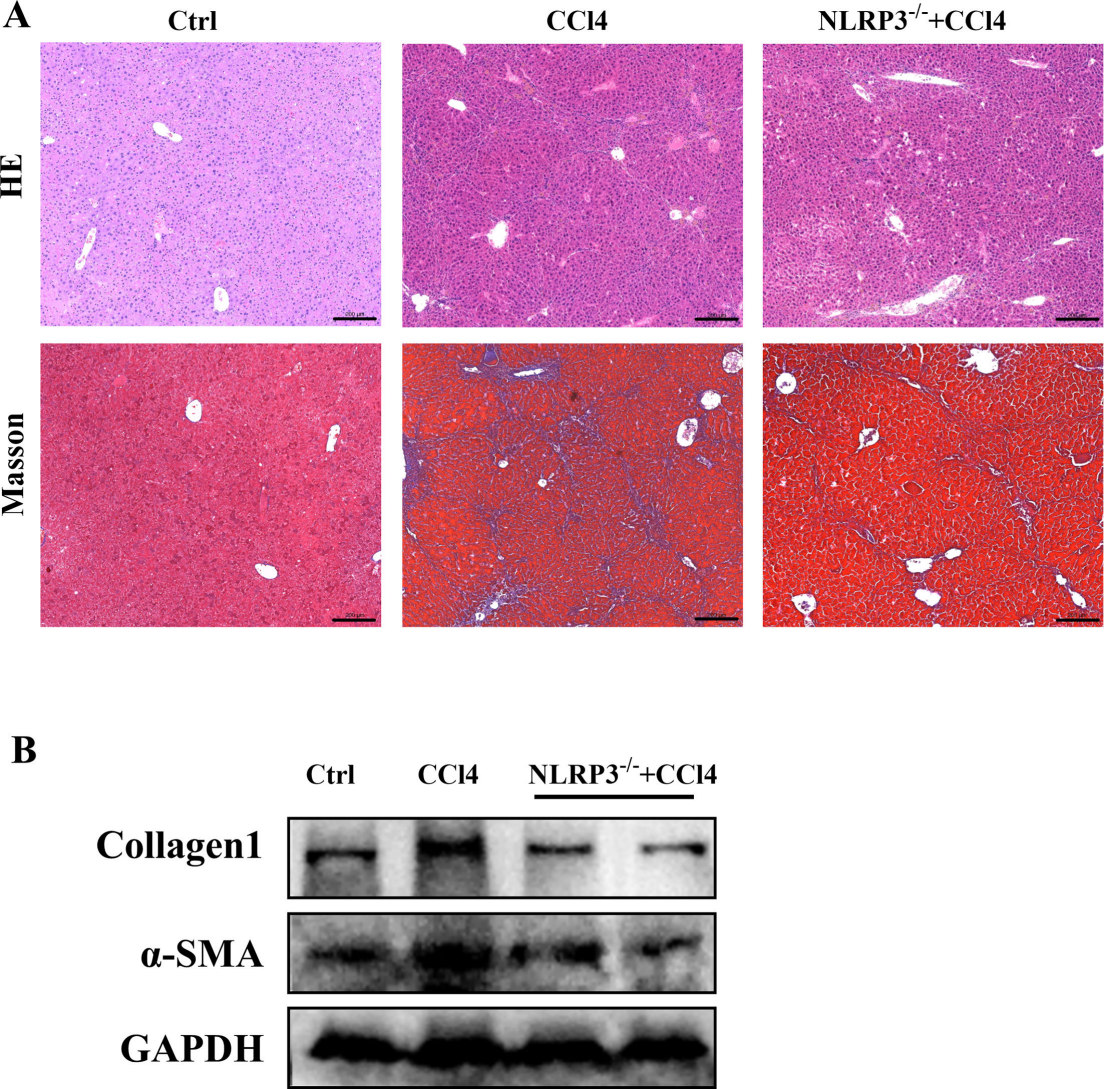
NLRP3 knockout alleviated liver fibrosis. (**A**) Representative images of liver stained with hematoxylin and eosin and Masson’s trichrome staining (200 µm). (**B**) Hepatic protein expression of α-SMA and collagen1.

Our previous study indicated that CCl4 promoted liver fibrosis in mice, and knockout of NLRP3 alleviated fibrosis (3), and current research also indicated that NLRP3 deletion attenuated CCl4-induced hepatic fibrosis (Fig. 1). The current study further explored the role of NLRP3 in BA metabolism and gut microbiota to understand the role of NLRP3 in liver fibrosis.

### Targeted metabolomics for liver BAs

**Fig 2:**
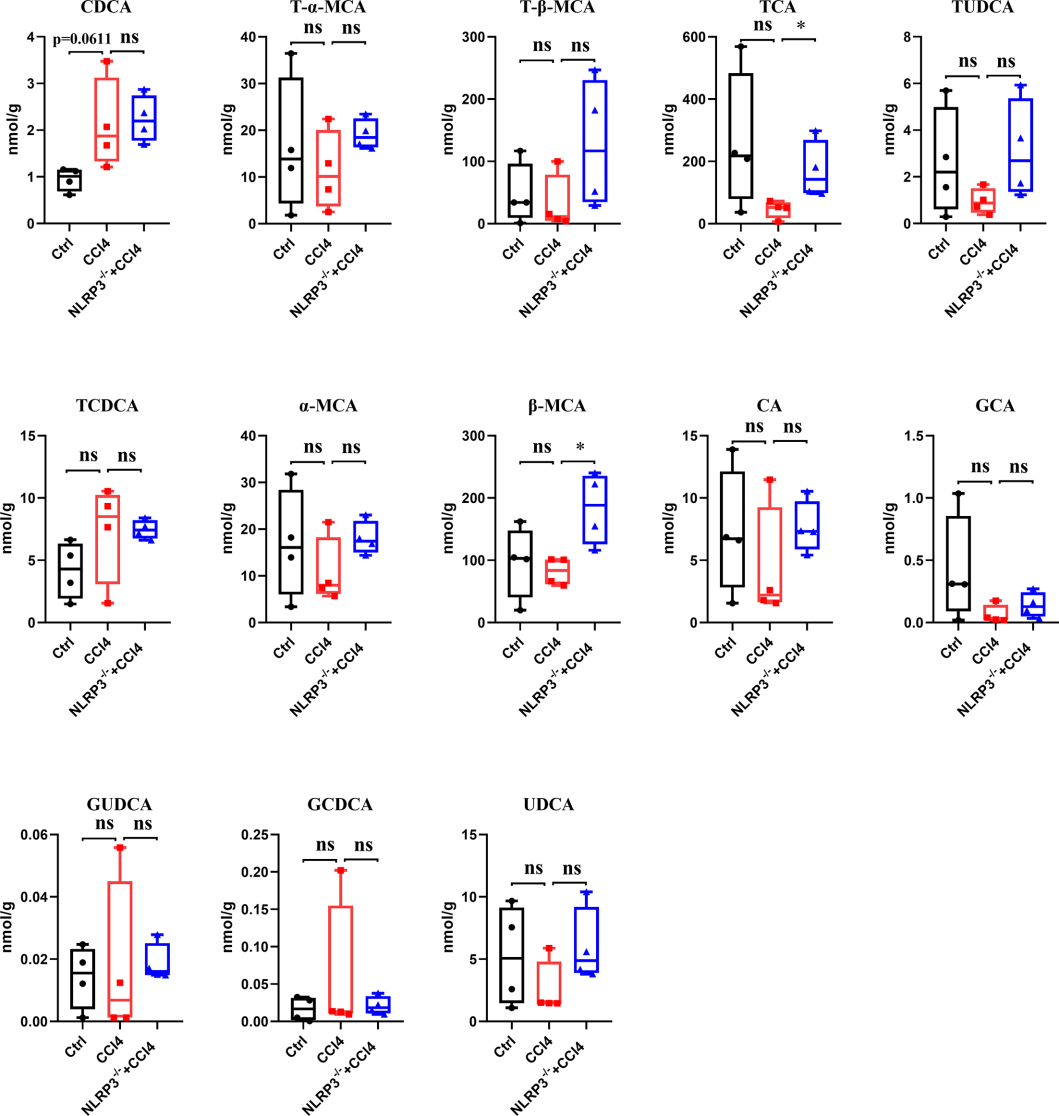
The liver primary bile acid profile among control, CCl4, and NLRP3^−/−^ + CCl4 mice. Statistical analysis was performed using two-tailed Student’s t test (normally distributed continuous data) or Mann-Whitney test (non-normally distributed continuous data). *P < 0.05. ns, not significant. The box plots showed the minimum to maximum values (whiskers).

The liver primary BA profile was determined (Fig. 2; Supplementary S1). CDCA and TCDCA showed an increased trend, while T-α-MCA, T-β-MCA, TCA, TUDCA, α-MCA, β-MCA, CA, GCA, GUDCA, and UDCA had a decreased trend in the CCl4 group compared to the control group. In the NLRP3^−/−^ +CCl4 mice, T-α-MCA, T-β-MCA, TCA, TUDCA, α-MCA, β-MCA, CA, GCA, GUDCA, and UDCA showed an increased trend compared to the CCl4 group. NLRP3 was the important active factor in inflammation and damage. These findings showed that the biosynthesis of primary BAs was reduced and knockdown of NLRP3 restored the biosynthesis of primary BAs in mice with CCl4-induced liver fibrosis.

**Fig 3:**
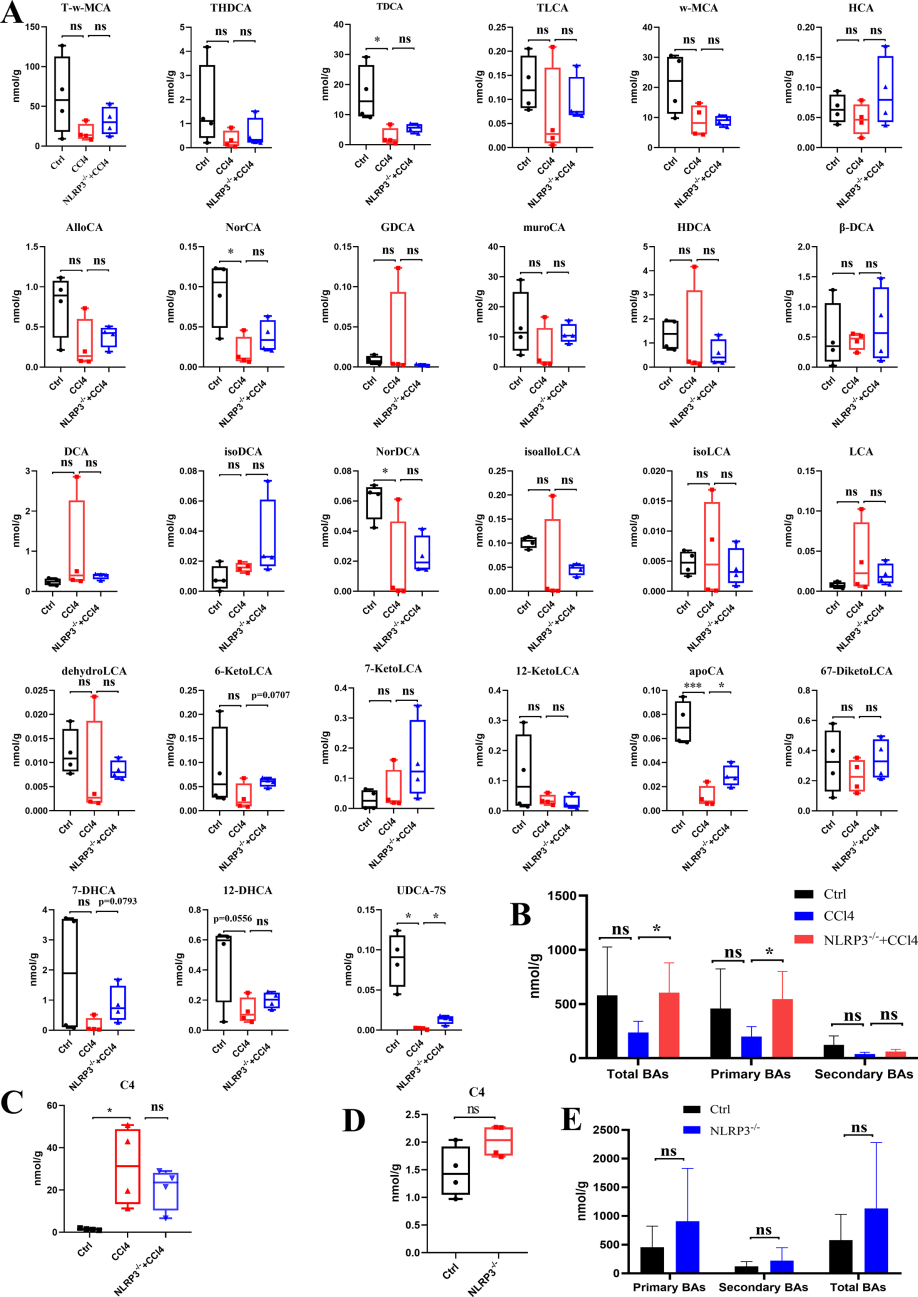
The liver secondary bile acid profile and C4 concentration among control, CCl4, and NLRP3^−/−^ +CCl4 mice. (**A**) The liver secondary bile acid profile among control, CCl4, and NLRP3^−/−^ +CCl4 mice. (**B**) The concentrations of total BAs, primary BAs, and secondary BAs in the liver among control, CCl4, and NLRP3^−/−^ +CCl4 mice. (**C**) The C4 concentration in the liver among control, CCl4, and NLRP3^−/−^ +CCl4 mice. (**D**) The C4 concentration in the liver between control and NLRP3^−/−^ mice. (**E**) The concentrations of total BAs, primary BAs, and secondary BAs in the liver between control and NLRP3^−/−^ mice. Statistical analysis was performed using two-tailed Student’s t test (normally distributed continuous data) or Mann-Whitney test (non-normally distributed continuous data). *P < 0.05. ***P < 0.001. ns, not significant. The box plots showed the minimum to maximum values (whiskers).

The liver secondary BA profile of the liver was determined (Fig. 3A; Supplementary S2). The levels of AlloCA, NorCA, muroCA, HDCA, NorDCA, isoalloLCA, dehydroLCA, 6-KetoLCA, apoCA, 6,7-diketoLCA, 12-DHCA, and UDCA-7S showed a decreased trend in CCl4-induced mice compared to the control group, while the levels of secondary BAs showed an increased trend in the NLRP3^−/−^ +CCl4 mice compared to CCl4-induced mice. Fig. 3B indicated that total BAs, primary BAs, and secondary BAs showed a decreasing trend in the CCl4 group compared to the control group, and NLRP3 knockout improved CCl4-induced BAs disorder. The C4 acts as an alternative pathway for bile acid synthesis in the liver. Fig. 3C indicated that increased C4 levels were observed in the CCl4 group and a decreased trend of C4 levels in NLRP3^−/−^ +CCl4 mice. Additionally, the levels of C4 and BAs showed no significant alterations in NLRP3^−/−^ mice compared with the control group (Fig. 3D and E; Supplementary S3). The results showed that NLRP3 knockout reverses the impairment of hepatic bile acid metabolism caused during CCl4-induced liver fibrosis.

### NLRP3 knockout altered the gut flora of CCl_4_-induced liver fibrosis

To better understand the effect of the NLRP3 knockout on the gut flora in mice, we extracted bacterial DNA from the intestinal contents and analyzed it using 16S rRNA sequencing.

First, we analyzed the impact of NLRP3 knockout on gut microbiota. Compared to the control group, the NLRP3^−/−^ group exhibited increased intestinal alpha diversity and altered beta diversity (Fig. S1 and Supplementary S4). Additionally, we observed changes in signaling pathway activity and gut microbiota-derived ECs (Fig. S2). These findings indicated NLRP3 played a critical regulatory role in gut microbiota.

**Fig 4:**
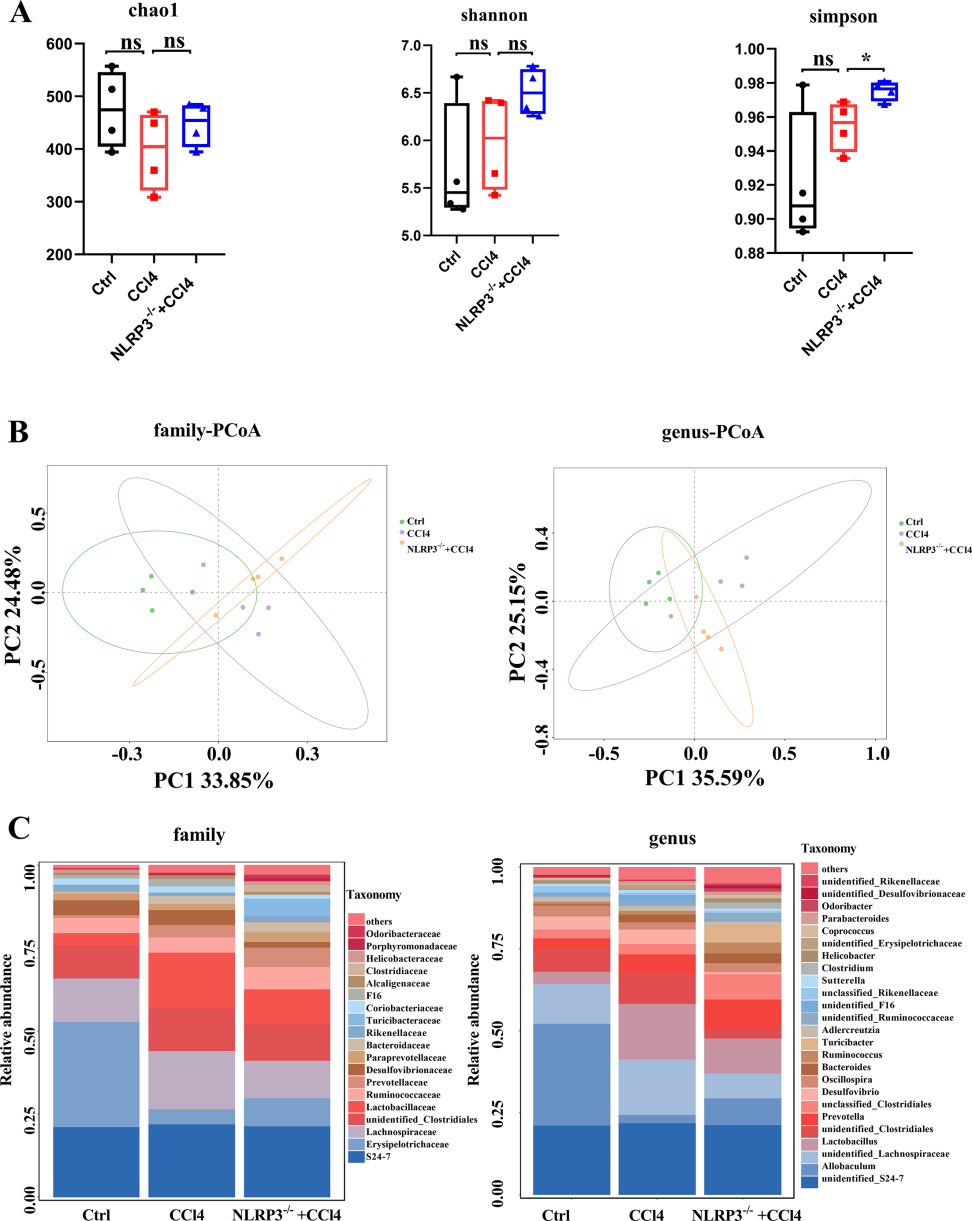
Alpha diversity and beta diversity of microbial community structure among control, CCl4, and NLRP3^−/−^ +CCl4 mice. (**A**) Alpha diversity indices of Chao1, Shannon, and Simpson. (**B**) Beta diversity of microbial community structure: PCoA based on Bray-Curtis distances at family and genus levels. (**C**) The relative abundances at family and genus levels. Statistical analysis was performed using two-tailed Student’s t test (normally distributed continuous data) or Mann-Whitney test (non-normally distributed continuous data). *P < 0.05. ns, not significant. The box plots showed the minimum to maximum values (whiskers).

**Fig 5:**
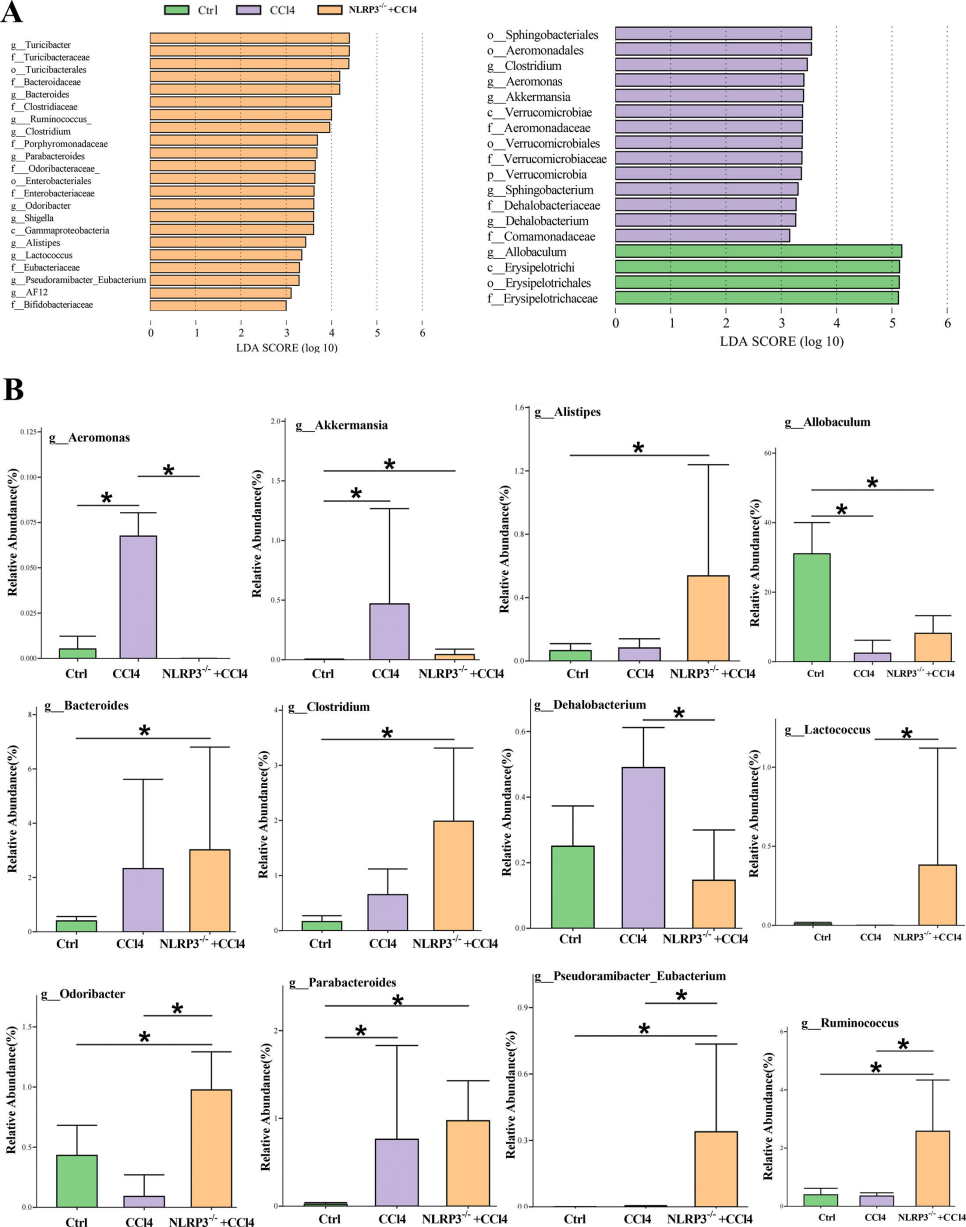
The differential gut microbiota among control, CCl4, and NLRP3^−/−^ +CCl4 mice. (**A**) The results of LEfSe analysis displayed the differential changed gut microbiota at different taxa levels among three groups (LDA ≥3.0). (**B**) The differential microbiota at the genus level among three groups. Statistical analysis was performed using two-tailed Student’s t test (normally distributed continuous data) or Mann-Whitney test (non-normally distributed continuous data). Data are expressed as the mean ± SD. *P < 0.05.

Next, we investigated the role of NLRP3 knockout in regulating gut microbiota alterations during CCl4-induced liver fibrosis. Alpha diversity showed that there was no significant difference in microbiota diversity based on Chao1 and Shannon indices among control, CCl4, and NLRP3^−/−^ + CCl4 groups. Simpson pointed out that NLRP3 knockout contributed to the diversity of the microbiota (Fig. 4A). In addition, PCoA was performed to analyze β diversity and showed that the distributions of gut microbiota among the three groups differed from one another at family and genus levels (Fig. 4B). As shown in Fig. 4C, the abundances at family and genus levels were different among the three groups (Supplementary S5). To analyze the variation in gut microbiota, LEfSe analysis was used. The results showed that the gut microbiota was differently abundant among the three groups (Fig. 5A). At the genus level, the abundances of Aeromonas, Akkermansia, and Dehalobacterium were increased in the gut of the CCl4 group and decreased in NLRP3^−/−^ +CCl4 mice. The abundances of Allobaculum, Odoribacter, and Ruminococcus decreased in the gut of CCl4 group and increased in NLRP3^−/−^+CCl4 mice. The abundances of Alistipes, Bacteroides, Clostridium, Lactococcus, Parabacteroides, and Pseudoramibacter_Eubacterium were significantly increased in NLRP3^−/−^+CCl4 mice (Fig. 5B).

### Relationships between LPS in liver tissue and the gut microbiota

**Fig 6:**
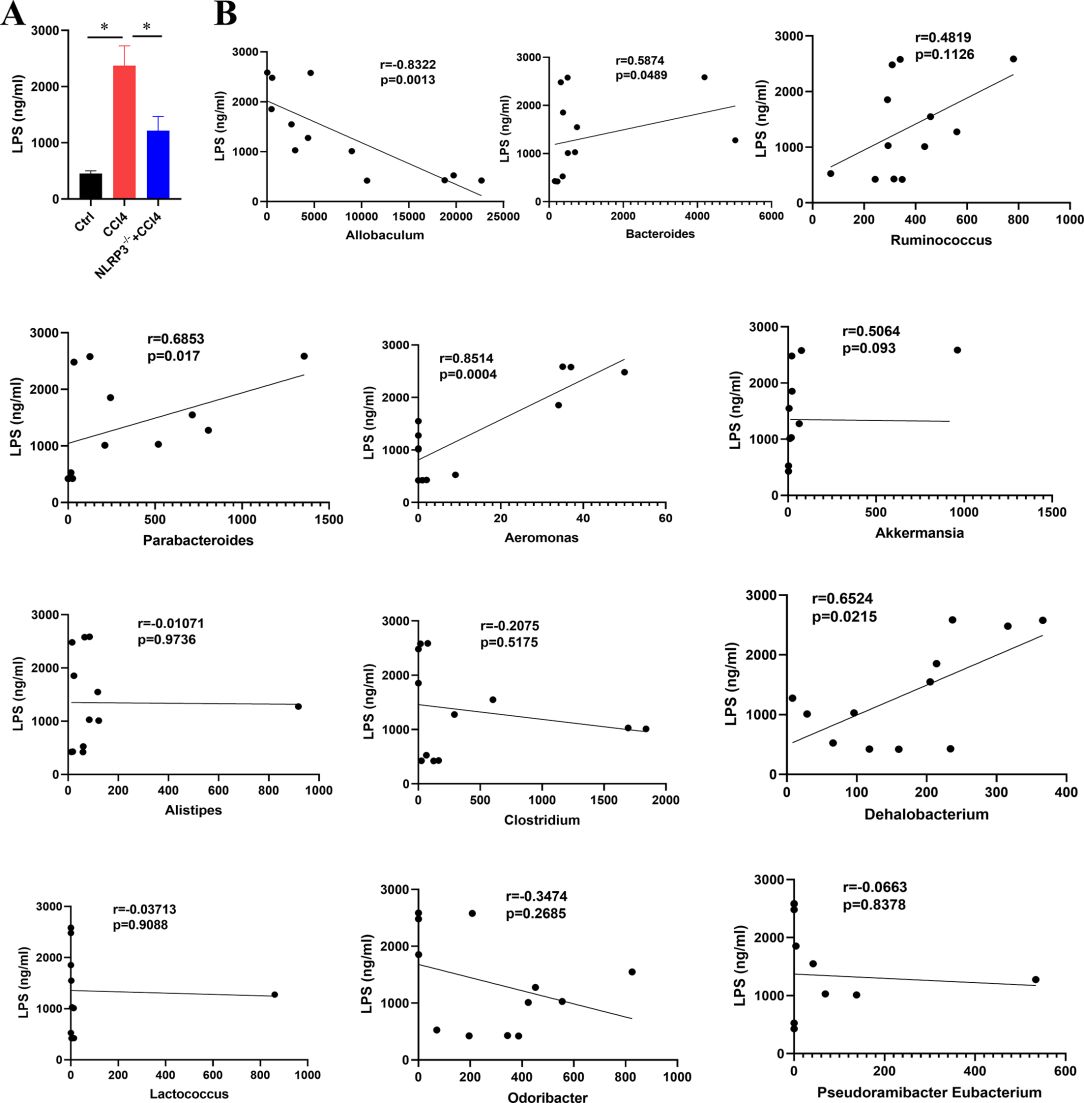
Correlation between LPS in liver tissues and differential gut microbiota. (**A**) LPS concentration in the liver tissues among control, CCl4, and NLRP3^−/−^ +CCl4 mice. Statistical analysis was performed using two-tailed Student’s t test (normally distributed continuous data) or Mann-Whitney test (non-normally distributed continuous data). Data are expressed as the mean ± SD. (**B**) Spearman correlations between LPS and differential gut microbiota. *P < 0.05.

Liver LPS concentration in the CCl4 group was significantly higher than in the control group. However, liver LPS concentration decreased in the NLRP3^−/−^ + CCl4 group (Fig. 6A). LEfSe-derived discriminant taxa were analyzed for associations with LPS concentrations. Allobaculum was negative with LPS, while Bacteroides, Parabacteroides, Aeromonas, and Dehalobacterium had a positive correlation with LPS (Fig. 6B).

### The effects of NLRP3 knockout on the signaling pathways of gut microbiota in CCl4-induced liver fibrosis

**Fig 7:**
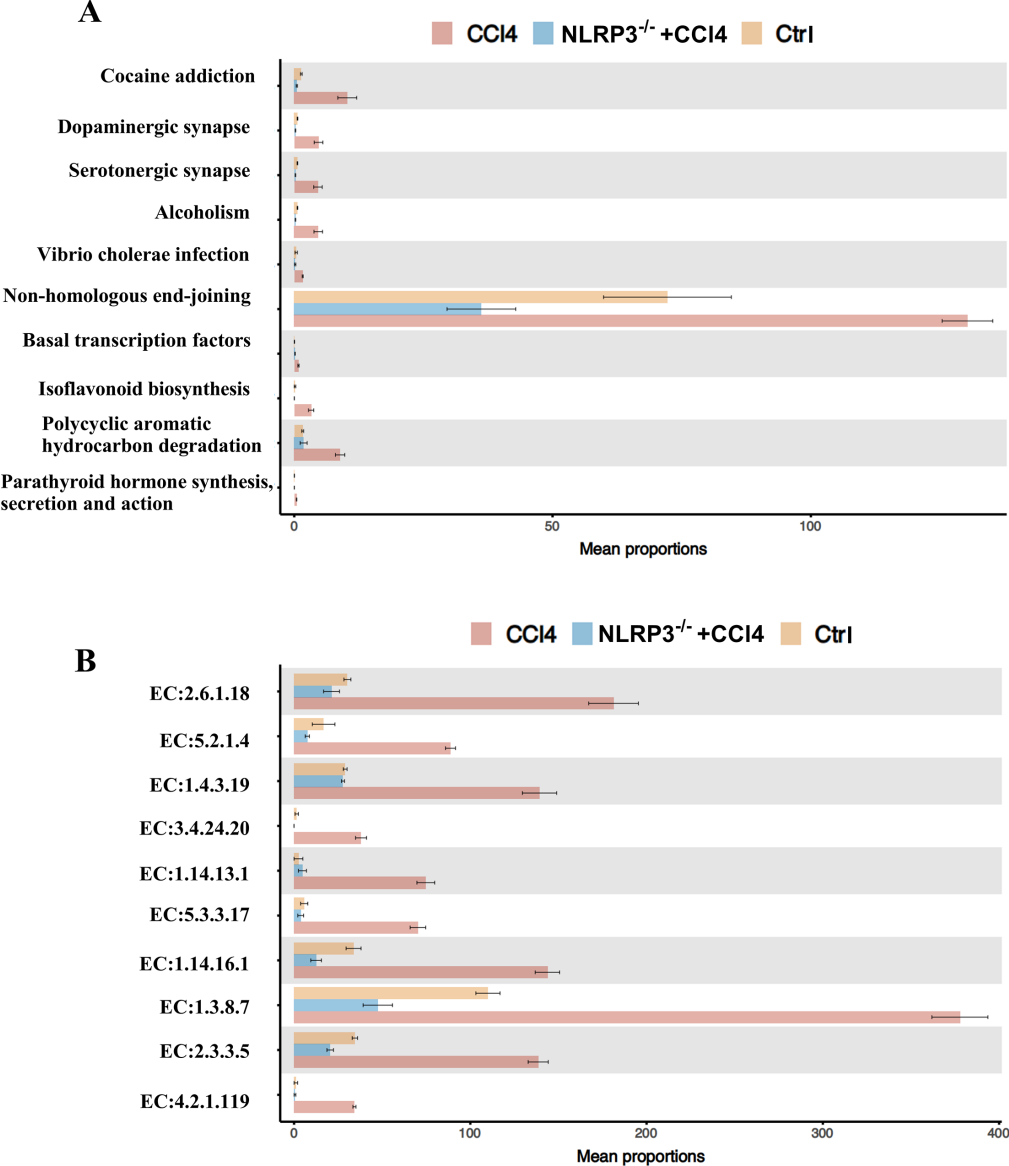
Differential metabolic pathways and differentially expressed enzymes among control, CCl4, and NLRP3^−/−^ +CCl4 mice. (**A**) Top ten differential metabolic pathways among control, CCl4, and NLRP3^−/−^ +CCl4 mice. (**B**) The ten most commonly differentially expressed enzymes among control, CCl4, and NLRP3^−/−^ +CCl4 mice.

Some important signaling pathways such as dopaminergic synapse, serotonergic synapse, non-homologous end joining, basal transcription factors, and isoflavonoid biosynthesis were upregulated in the CCl4 group and reversed by NLRP3 knockout (Fig. 7A). The top 10 enzymes, namely, EC 2.6.1.18 (beta-alanine--pyruvate transaminase), EC 5.2.1.4 (maleylpyruvate isomerase), EC 1.4.3.19 (glycine oxidase), EC 3.4.24.20 (peptidyl-Lys metalloendopeptidase), EC 1.14.13.1 (salicylate 1-monooxygenase), EC 2.3.3.5 (2-methylcitrate synthase), EC 1.14.16.1 (phenylalanine 4-monooxygenase), EC 1.3.8.7 (medium-chain acyl-CoA dehydrogenase), EC 4.2.1.119 (enoyl-CoA hydratase 2), and EC 5.3.3.17 (trans-2,3-dihydro-3-hydroxyanthranilate isomerase) were upregulated in the CCl4 group and reversed by NLRP3 knockout (Fig. 7B). The altered structure of gut microbiota in the CCl4 group and NLRP3^−/−^+CCl4 mice suggested that NLRP3 might exert its pro-inflammatory role via altering the activity of signaling pathways and ECs in the gut microbiota to facilitate the development of liver fibrosis.

### Relationships between the gut microbiota or LPS and liver BA metabolism

**Fig 8:**
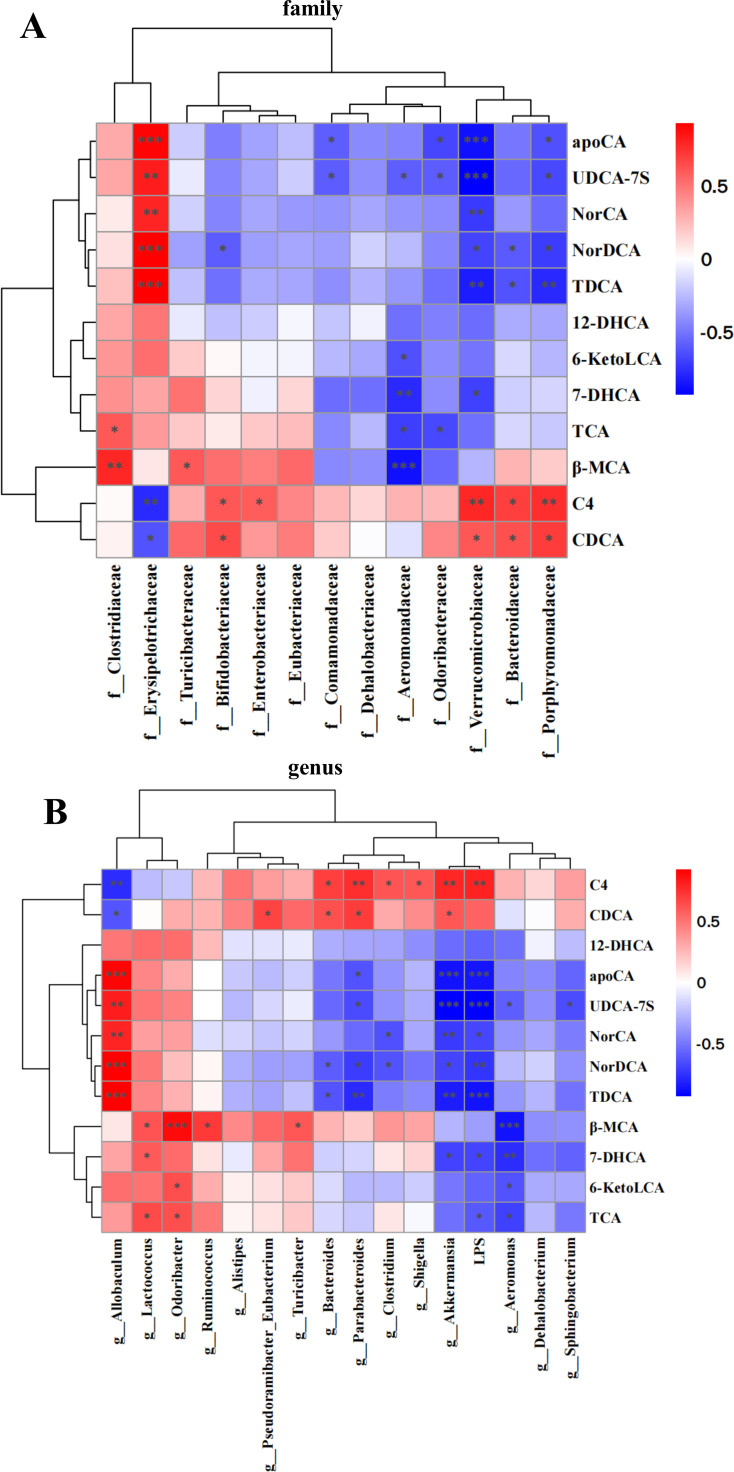
The correlations between the differential gut microbiota or LPS and liver bile acids. (**A**) The correlations between the differential gut microbiota at family level and differential liver bile acids. (**B**) The correlations between the differential gut microbiota at genus level or LPS and differential liver bile acids. *P < 0.05. **P < 0.01. ***P < 0.001.

At the family level, *Verrucomicrobiaceae, Bifidobacteriaceae, Porphyromonadaceae,* and Bacteroidaceae were positively correlated with the concentrations of CDCA and C4, and Verrucomicrobiaceae was negatively correlated with the concentrations of UDCA-7S, TDCA, NorDCA, NorCA, apoCA, and 7-DHCA. The abundance of Turicibacteraceae was positively correlated with the content of β-MCA. The abundance of Porphyromonadaceae was negatively correlated with the contents of UDCA-7S, TDCA, NorDCA, and apoCA. Odoribacteraceae was negatively correlated with the contents of UDCA-7S, TCA, and apoCA. Erysipelotrichaceae was positively correlated with the contents of UDCA-7S, TDCA, NorDCA, NorCA, and apoCA, and was negatively correlated with the contents of CDCA and C4. Enterobacteriaceae was positively correlated with the content of C4. Comamonadaceae was negatively correlated with the contents of UDCA-7S and apoCA. Clostridiaceae was positively correlated with the contents of β-MCA and TCA. Bifidobacteriaceae was negatively correlated with the contents of NorDCA. Bacteroidaceae was negatively correlated with the contents of TDCA and NorDCA. Aeromonadaceae was negatively correlated with the contents of β-MCA, UDCA-7S, TCA, 7-DHCA, and 6-ketoLCA. (Fig. 8A).

At the genus level, Turicibacter was positively correlated with the content of β-MCA. Sphingobacterium was negatively correlated with the content of UDCA-7S. Shigella was positively correlated with the content of C4. Ruminococcus was positively correlated with the content of β-MCA. Pseudoramibacter_Eubacterium was positively correlated with the content of CDCA. Parabacteroides, Akkermansia, and Bacteroides were positively correlated with the contents of C4 and CDCA, and Parabacteroides was negatively correlated with the contents of TDCA, apoCA, UDCA-7S, and NorDCA. Odoribacter was positively correlated with the contents of TCA, β-MCA, and 6-ketoLCA. Lactococcus was positively correlated with the contents of TCA, β-MCA, and 7-DHCA. Clostridium was positively correlated with the content of C4 and was negatively correlated with the contents of NorCA and NorDCA. Bacteroides was negatively correlated with the contents of TDCA and NorDCA. Allobaculum was positively correlated with the contents of TDCA, NorCA, apoCA, UDCA-7S, and NorDCA and was negatively correlated with the contents of C4 and CDCA. Akkermansia was negatively correlated with the contents of TDCA, 7-DHCA, NorCA, apoCA, UDCA-7S, and NorDCA. Aeromonas was negatively correlated with the contents of TCA, β-MCA, 7-DHCA, 6-ketoLCA, and UDCA-7S. LPS showed a positive correlation with C4, but negative correlations with apoCA, UDCA-7S, NorCA, NorDCA, TDCA, 7-DHCA, and TCA (Fig. 8B).

## DISCUSSION

BAs are important liver metabolites. Studies also showed that the BAs promoted liver fibrosis (9, 11, 12). Numerous studies have reported that activation of NLRP3 induced extensive extracellular matrix production from hepatic stellate cells and promoted the development of fibrosis (13–15). However, it is unclear whether NLRP3 has an impact on BA metabolism in liver fibrosis. As shown in Fig. 3B, total BAs, primary BAs, and secondary BAs were significantly reduced in the CCl4 group compared to controls, indicating impaired BA synthesis due to hepatocyte injury. NLRP3 knockout restored BA levels, demonstrating its hepatoprotective effect against fibrosis. Since BAs are exclusively synthesized in hepatocytes, their decline directly reflects cellular dysfunction. Interestingly, although intrahepatic BA production decreased (Fig. 3B), clinical studies reported elevated serum BA levels in advanced fibrosis, likely due to disrupted biliary excretion (16). This discrepancy underscores the complexity of BA metabolism in fibrosis. Most studies have focused on serum/fecal BA profiles, while hepatic BA metabolism in fibrotic livers remains poorly characterized due to the difficulty of obtaining tissue samples. Our study provided direct evidence of intrahepatic BA alterations. Nevertheless, additional investigations are warranted to examine alterations in intrahepatic bile acids and their mechanistic role in the progression of liver fibrosis.

Cyp7a1 is the rate-limiting enzyme that catalyzes the decomposition of cholesterol into bile acid in the liver (17). The increased level of 7α-hydroxy-4-cholesten-3-one (C4) reflects the activated function of Cyp7a1 (18). It has been reported that low production of bile acids in the liver indicates rapid worsening of primary sclerosing cholangitis (19), suggesting that bile acid content is important for liver function. In the present study, the C4 level increased in the CCl4 group and decreased by NLRP3 knockout, but the total bile acid content decreased in the CCl4 group and increased by NLRP3 knockout. A possible explanation is that C4 reflected the compensatory repair of hepatocytes.

Numerous studies have shown that the imbalance between BAs and gut bacteria promotes liver fibrosis (20–24). CCl4 reduced the formation of BAs and resulted in fewer BAs entering the gut, which can make a significant difference in gut bacteria. In our study, each of the three groups (control group, CCl4 group, and NLRP3^−/−^ + CCl4 group) has a different composition when it comes to gut microbiota analysis. The gut microbiota of mice in the CCl4 group contained fewer beneficial bacteria and more harmful bacteria compared to the control group. The trends in the NLRP3^−/−^ + CCl4 group were different from those in the CCl4 group. Ruminococcus showed a significant negative association with fibrosis severity only in non-obese subjects (25). In the current study, the abundance of Ruminococcus was remarkably increased in the NLRP3^−/−^ group. *Zanthoxylum bungeanum* amides increased the abundance of Allobaculum, which produces short-chain fatty acids and may activate AMPK/Nrf2 signaling to ameliorate nonalcoholic fatty liver disease (26). In the current study, NLRP3 knockout improved Allobaculum abundance, decreased in the CCl4 group, and LPS in liver was negatively correlated with Allobaculum. Aeromonas has been reported to be associated with liver cirrhosis (27). The abundance of Akkermansia bacteria was significantly increased in the CCl4 group and was positively associated with AST and ALT (24). In the current study, the abundances of Aeromonas and Akkermansia were increased in the CCl4 group and decreased in the NLRP3^−/−^ group. It was reported that Bacteroides and Parabacteroides were more abundant in CCl4 mice, and *Swertia bimaculata* ameliorated CCl4-induced liver injury and reduced the abundances of Bacteroides and Parabacteroides (28). In the current study, the abundances of Bacteroides and Parabacteroides were increased in the CCl4 group, but NLRP3 knockout did not decrease their abundance, suggesting that NLRP3 had no significant effect on Bacteroides and Parabacteroides. We also discovered that liver LPS concentration was positively correlated with the abundances of Bacteroides and Parabacteroides, suggesting that Bacteroides and Parabacteroides exerted their fibrosis-promoting roles in CCl4-induced liver fibrosis. A lower abundance of Odoribacter, a bacterium with high production of short-chain fatty acids, was found in non-alcoholic fatty liver disease compared to healthy subjects (29). Odoribacter was in a low abundance in the CCl4 group and recovered a higher abundance after NLRP3 knockout in the current study. Taken together, NLRP3 may also exert its pro-fibrosis role by disturbing the balance of gut microbiota.

In the hepatic encephalopathy caused by cholestasis, the dopaminergic and serotonergic pathways were disturbed (30), which can lead to behavioral disorders. Cholestasis triggers liver fibrosis. Fig. 7A indicated that dopaminergic synapse and serotonergic synapse signaling pathways were upregulated in the CCl4 group and reversed by NLRP3 knockout. It suggested that CCl4-induced fibrosis mice may have behavioral defects. CCl4 may induce DNA double-strand breaks, which are the most dangerous type of DNA damage. Non-homologous end joining is responsible for the repair of DNA double-strand breaks (31). We found that the non-homologous end joining pathway was upregulated in the CCl4 group, suggesting that the body repair function was activated. The biosynthesis of isoflavonoid was upregulated in the CCl4 group and reversed by NLRP3 knockout. Isoflavonoid is a group of water-soluble flavones that are antioxidants (32). Due to the body’s defense, the increased isoflavonoid biosynthesis in the CCl4 group may be important to alleviate the toxicity of CCl4. Figure 7B showed that the activity of ten enzymes was upregulated in the CCl4 group and reversed by NLRP3 knockout. These results indicated that NLRP3 may exert its pro-inflammatory role via altering the activity of signaling pathways and ECs in the gut microbiota.

Due to the interaction between BAs and gut microbiota, probiotic interventions have important prospects for the treatment of liver fibrosis. *Lactobacillus rhamnosus R0011* alleviated liver injury in rats with CCl4-induced liver fibrosis by protecting the intestinal barrier and restoring microbial health (33). *Lactiplantibacillus plantarum* LPJZ-658 improved BA metabolism and gut microbiota dysbiosis to mitigate NASH (34). Some probiotic microbiota have shown effectiveness in treating liver fibrosis. In the current study, Allobaculum was abundant in control mice and remarkably reduced in the CCl4 group compared to other microbiota. NLRP3 knockout increased the Allobaculum abundance. It suggested that Allobaculum was a potentially protective microbiome for alleviating liver fibrosis.

While our study identifies NLRP3 as a key modulator of bile acid and microbiota in liver fibrosis, several limitations warrant consideration. First, the correlative nature of our findings requires validation through mechanistic experiments (e.g., fecal microbiota transplantation and bile acid supplementation). Second, the small cohort size may underpower statistical detection of subtle effects. Finally, the discordance between hepatic bile acid reduction and classical fibrotic bile acid accumulation merits further investigation. Future studies integrating metagenomics and targeted metabolite profiling will clarify these interactions.

### Conclusion

The reduced BA level in CCl4-induced liver fibrosis reflected liver injury. NLRP3 knockout restored BA levels, and NLRP3 knockout was shown to protect the liver from damage. Elevated LPS reflected bacterial translocation into the liver. NLRP3 knockout ameliorated CCl4-induced gut microbiota dysregulation. Taken together, NLRP3 damages the liver, reduces BA production, and could induce bacterial translocation into the liver to promote liver fibrosis.

## Data Availability

The data generated and/or analyzed are available from the corresponding author on reasonable request.

## SUPPLEMENTAL MATERIAL

Data S1 spectrum.00148-25-s0001.xlsx Primary BA profile in the liver of control, CCl4, and NLRP3^−/−^ + CCl4 mice.

Data S2 spectrum.00148-25-s0002.xlsx Secondary BA profile and C4 concentration in the liver of control, CCl4, and NLRP3^−/−^ + CCl4 mice.

Data S3 spectrum.00148-25-s0003.xlsx Primary BA profile, secondary BA profile, and C4 concentration in the liver of control and NLRP3^−/−^ mice.

Data S4 spectrum.00148-25-s0004.xlsx The abundances of gut microbiota at the family and genus levels in control and NLRP3^−/−^ mice.

Data S5 spectrum.00148-25-s0005.xlsx The relative abundances of gut microbiota at family and genus level in control, CCl4, and NLRP3^−/−^ + CCl4 mice.

Figure S1 spectrum.00148-25-s0006.tif Alpha diversity and beta diversity of microbial community structure between control and NLRP3^−/−^ mice.

Figure S2 spectrum.00148-25-s0007.tif Differential metabolic pathways and differentially expressed enzymes between control and NLRP3^−/−^ mice.

ASM does not own the copyrights to Supplemental Material that may be linked to, or accessed through, an article. The authors have granted ASM a non-exclusive, world-wide license to publish the Supplemental Material files. Please contact the corresponding author directly for reuse.
