# Supplementary figures and images for: Role of NLRP3 in the metabolism of bile acids and gut microbiota in CCl4-induced liver fibrosis
Source: Microbiol Spectr. 2025 Jul 21;13(9):e00148-25. doi: 10.1128/spectrum.00148-25 (PMC12403853; doi:10.1128/spectrum.00148-25)

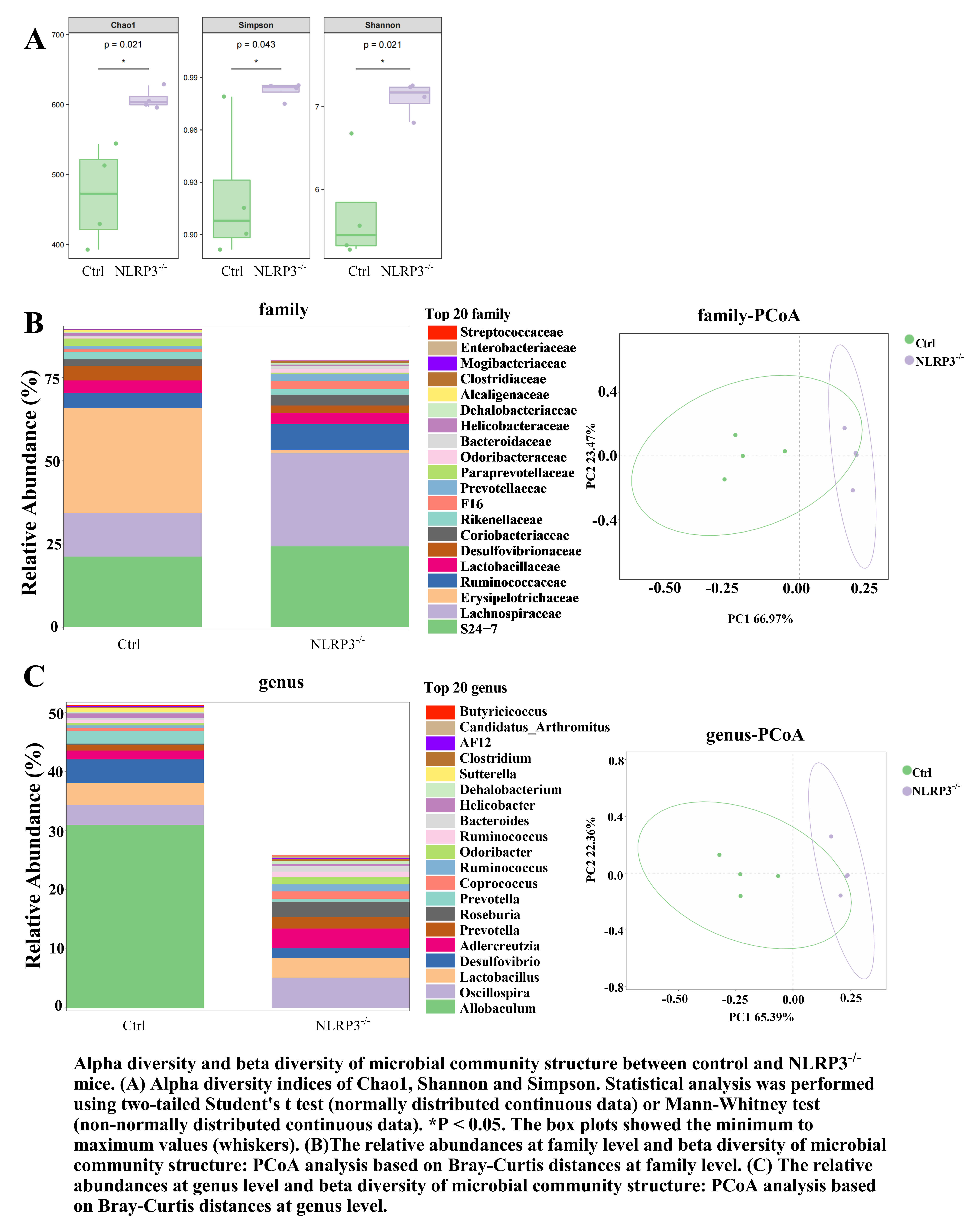

Supplement: Figure S1 — Alpha diversity and beta diversity of microbial community structure between control and NLRP3−/− mice. [file spectrum.00148-25-s0006.tif]

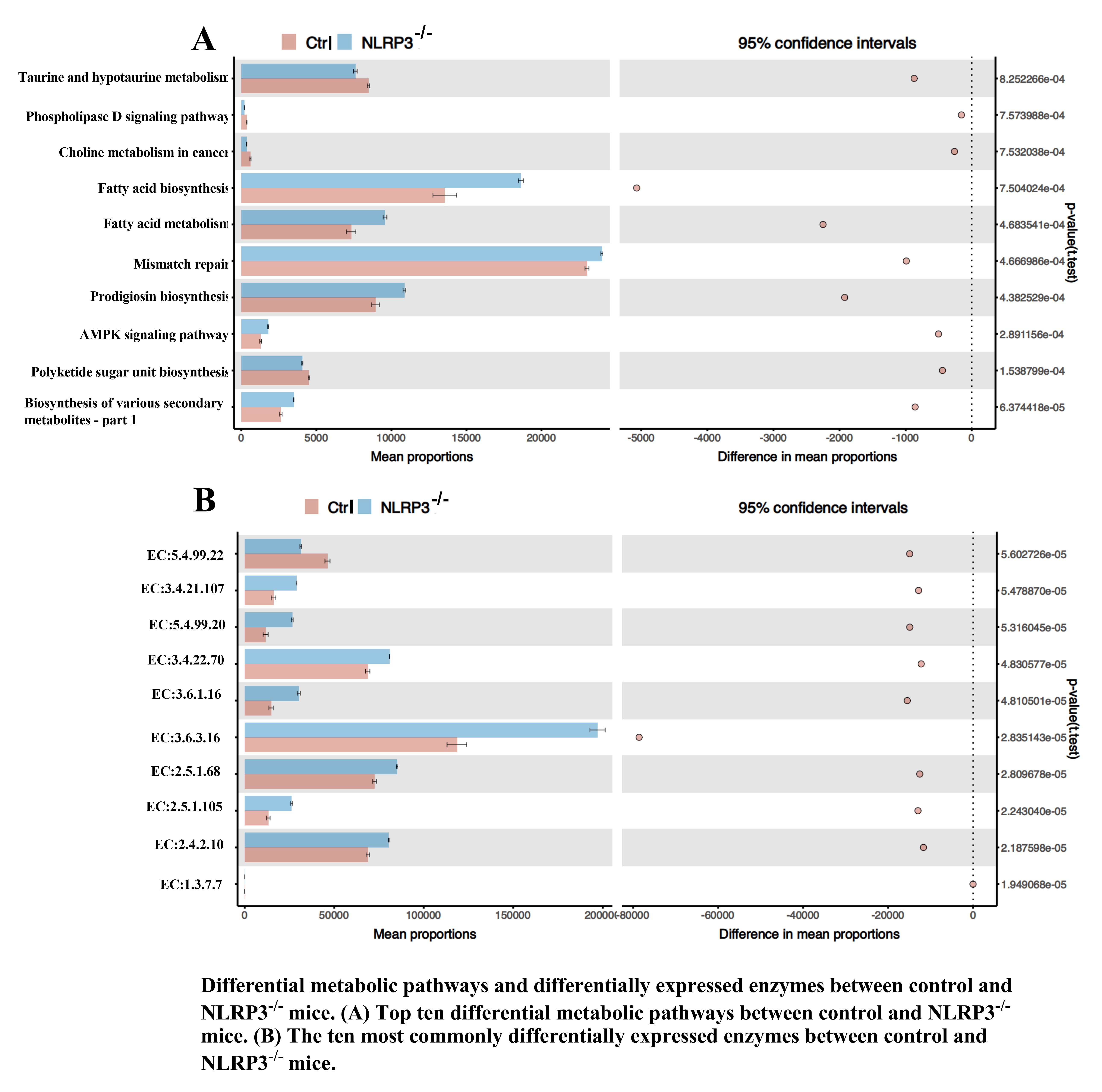

Supplement: Figure S2 — Differential metabolic pathways and differentially expressed enzymes between control and NLRP3−/− mice. [file spectrum.00148-25-s0007.tif]
